# Uncertainty Calculation for Spectral-Responsivity Measurements

**DOI:** 10.6028/jres.114.020

**Published:** 2009-10-01

**Authors:** John H Lehman, CM Wang, Marla L Dowell, Joshua A Hadler

**Affiliations:** National Institute of Standards and Technology, Boulder, CO 80305

**Keywords:** laser, monochromator, optical fiber, optical power, spectral responsivity, uncertainty calculation

## Abstract

This paper discusses a procedure for measuring the absolute spectral responsivity of optical-fiber power meters and computation of the calibration uncertainty. The procedure reconciles measurement results associated with a monochromator-based measurement system with those obtained with laser sources coupled with optical fiber. Relative expanded uncertainties based on the methods from the Guide to the Expression of Uncertainty in Measurement and from Supplement 1 to the “Guide to the Expression of Uncertainty in Measurement”-Propagation of Distributions using a Monte Carlo Method are derived and compared.

An example is used to illustrate the procedures and calculation of uncertainties.

## 1. Introduction

The National Institute of Standards and Technology (NIST) provides a calibration service for absolute spectral-responsivity of optical-fiber power meters at wavelengths between 400 nm and 1800 nm [1]. The service is unique because it characterizes the optical-fiber power meter, not just an optical detector, which is normally coupled to a laser with an optical fiber. We rely on two measurement systems for a single calibration; one system to provide complete wavelength coverage, the second to duplicate the systematic behavior of coherent light exiting the fiber. In the first instance, we rely on a broadband source and a monochromator (the *monochromator system*) to cover the entire wavelength range because a continuously variable laser source is not currently available. In the second instance, several laser-diode sources, independently coupled to optical fiber (the *fiber system*), provide the optical input for measurements at discrete wavelengths. The reference detector for the monochromator system is a pyroelectric wedge-trap detector and the reference detector for the fiber system is an electrically calibrated pyroelectric described elsewhere [2].

**Fig. 1:**
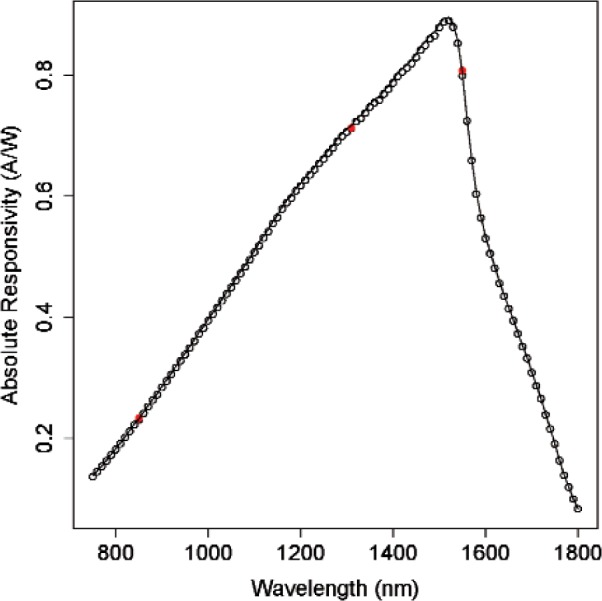
Spectral-responsivity measurements of a commercially available optical fiber power meter based on a 5 mm diameter, germanium photodiode. The empty circles are obtained from the monochromator system. The solid circles are results from the fiber system. The solid line represents the meter’s spectral responsivity calibration.

Figure 1 displays typical measurement results from the monochromator system and the fiber system. Measurements from the monochromator system consist of the meter’s relative responsivity (A/W) at 10 nm increments (transmitted through air) over the entire wavelength range of the meter. Measurements from the fiber system represent the absolute responsivity at several laser wavelengths, typically at 850 nm, 1310 nm, and 1550 nm. In each calibration procedure, measurements from the monochromator system are normalized to higher accuracy measurements made with the fiber system. That is, the results from the mono-chromator-based measurement are *adjusted* (multiplied by a constant) so that they agree as closely as possible with the fiber-based measurements. The uncertainty of the meter’s calibration depends on the disagreement between the fiber-based and the adjusted mono-chromator-based results, as well as the uncertainties of the independent measurements. Independent uncertainty analysis of the fiber-based and monochromator-based measurements, including various type A and type B evaluations of uncertainties due to the working standard and optical sources, is given in [1]. In the present work, we describe procedures for calculating the normalized spectral responsivity and its uncertainty at each wavelength increment. The procedures consist of a least-squares estimation of the adjustment and a ratio estimation of the spectral responsivity. We compare two methods for calculating the uncertainty of the normalized spectral responsivity. We use an example to illustrate the procedures.

## 2. Measurement Adjustment

Determination of the adjustment is based on the common spectral-responsivity measurements made at selected laser wavelengths. Let *k* be the number of laser wavelengths used, and (*x_i_*, *y_i_*), *i* = 1, …, *k*, be the fiber-based (*x_i_*) and monochromator-based (*y_i_*) measurements at these laser wavelengths. Since the measurement errors in *x_i_* are negligible relative to the measurement errors in *y_i_*, an adjustment factor *c* can be obtained by minimizing the sum of squares

$$\sum_{i=1}^{k}{\left(y_{i}-cx_{i}\right)}^{2}$$

and is given by

$$c=\frac{\sum_{i=1}^{k}x_{i}y_{i}}{\sum_{i=1}^{k}x_{i}^{2}}.$$

Once *c* is obtained, the monochromator-based measurements *y_j_* are normalized by

$${\tilde{y}}_{j}=y_{j}/c.$$ (1)

Also, based on the standard regression analysis, the square of the standard uncertainty of *c* is given by

$$u^{2}\left(c\right)=\frac{\sum_{i=1}^{k}{\left(y_{i}-cx_{i}\right)}^{2}}{\left(k-1\right)\sum_{i=1}^{k}x_{i}^{2}},$$

with *ν_c_* = *k* − 1 degrees of freedom.

## 3. Uncertainty Analysis

When one calculates the uncertainty of a ratio estimate like *ỹ_j_* in (1), two distinct scenarios must be recognized. The first involves dependent measurements where the quantities in the numerator and denominator are measured in the course of a single measurement experiment. In this scenario, it is expected that common sources of error exist that contribute to errors in estimating both quantities. Hannig et al. [3] calculated the uncertainty of the ratio estimate of dependent measurements based on the method proposed in the ISO *Guide to the Expression of Uncertainty in Measurement* (GUM) [4] and used it to construct confidence intervals. They compared the resulting GUM intervals with intervals obtained from an exact method, known as Fieller’s method [5], and concluded that the GUM interval is very similar to the exact Fieller interval and can be recommended in metrological applications.

For measurement experiments where the quantities in the numerator and denominator are uncorrelated, as in the case of *ỹ_j_* in this application, there is no exact interval available. (The reason they are not correlated is that *c* is determined from the measurements at laser wavelengths, while the *y_j_* used in calculating *ỹ_j_* are the monochromator-based measurements at non-laser wavelengths.) In this paper we first consider the GUM approach and calculate the uncertainty of *ỹ_j_* as

$$u^{2}\left({\tilde{y}}_{j}\right)=\frac{u^{2}\left(y_{j}\right)}{c^{2}}+\frac{y_{j}^{2}u^{2}\left(c\right)}{c^{4}},$$

where *u*(*y_j_*) is the combined standard uncertainty of the monochromator-based measurement *y_j_*, which is discussed in detail in [1]. To obtain the expanded uncertainty we need to obtain the *effective* degrees of freedom associated with *u* (*ỹ_j_*). The GUM recommends the use of the Welch-Satterthwaite formula to evaluate the degrees of freedom, which is given by

$$\nu =\frac{{\left(\frac{u^{2}(y_{j})}{c^{2}}+\frac{y_{j}^{2}u^{2}(c)}{c^{4}}\right)}^{2}}{\frac{u^{4}(y_{j})}{c^{4}\nu_{b}}+\frac{y_{j}^{4}u^{4}(c)}{c^{8}\nu_{c}}},$$

where *ν_b_* is the degrees of freedom corresponding to *u*(*y_j_*).

In spectral-responsivity measurements, a single relative uncertainty is calculated and reported for the entire measurement curve due to the multiplicative nature of the measurement equation [1]. That is, if we use the symbol *u_r_*(*y_j_*) to denote the relative uncertainty of *y_j_*, then

$$u_{r}(y_{j})=\frac{u(y_{j})}{y_{j}}=b,$$

where *b* is a constant and does not depend on index *j*. Similarly, the relative uncertainty of *c* is

$$u_{r}(c)=\frac{u(c)}{c}.$$

As a consequence, we obtain the relative uncertainty of the normalized measurement *ỹ_j_* as

$$u_{r}^{2}({\tilde{y}}_{j})=u_{r}^{2}(y_{j})+u_{r}^{2}(c),$$ (2)

and its associated effective degrees of freedom,

$$\nu =\frac{{\left(u_{r}^{2}(y_{j})+u_{r}^{2}(c)\right)}^{2}}{u_{r}^{4}(y_{j})/\nu_{b}+u_{r}^{4}(c)/\nu_{c}}.$$ (3)

The relative *expanded* uncertainty of *ỹ_j_* is then given by

$$U_{r}=t_{0.975,\nu }\sqrt{u_{r}^{2}(y_{j})+u_{r}^{2}(c),}$$ (4)

where *t*_0.975_, *_ν_* is the 0.975 quantile of the *t* distribution with *ν* degrees of freedom.

The Welch-Satterthwaite approximation in (3) is known to be effective when the two degrees of freedom *ν_b_* and *ν_c_* are of about the same order of magnitude [6]. Since in our calibration experiments, the number of laser wavelengths used is typically equal to three, i.e., *ν_c_* = 2, and *ν_b_* is much larger than 2, we also consider two alternative methods for calculating the relative expanded uncertainty of *ỹ_j_*. The first alternative method we consider is a fiducial procedure [7]-[11], and the second is the method from *Supplement 1* to the GUM [12]. For the current application, these two alternative methods produce identical results, so we will discuss only the Supplement 1 method here.

The Supplement 1 method obtains a probability density function (pdf) for the measurand by propagating the pdf’s of the input quantities appearing in the measurement equation.

The resulting pdf describes one’s knowledge of the measurand given the observed data and assumptions made in assigning the joint pdf of the input quantities used in propagation. Once the pdf is obtained, a 95 % uncertainty interval for the measurand can be constructed by finding two limits such that the area under the pdf between these limits is 95 %. For example, the limits can be the 0.025 and 0.975 quantiles of the distribution. The measurand for this problem is the true spectral responsivity at each wavelength. Using the Supplement 1 approach, it can be shown that the pdf of the measur-and is equivalent to the distribution of

$$Y_{j}=\frac{y_{j}-u(y_{j})T_{\nu_{b}}^{\left(1\right)}}{c-u(c)T_{\nu_{c}}^{\left(2\right)}},$$

where $T_{\nu_{b}}^{\left(1\right)}$ and $T_{\nu_{c}}^{\left(2\right)}$ are independent random variables from the *t* distributions with *ν_b_* and *ν_c_* degrees of freedom, respectively. We can also express *Y_j_* using the relative uncertainties of *y_j_* and *c* as

$$Y_{j}=\frac{1-u_{r}(y_{j})T_{\nu_{b}}^{\left(1\right)}}{1-u_{r}(c)T_{\nu_{c}}^{\left(2\right)}}{\tilde{y}}_{j}.$$ (5)

Let *z*_1_ and *z*_2_ be the 0.025 and 0.975 quantiles of the distribution of *Y_j_*; then (*z*_1_, *z*_2_) is a 95 % uncertainty interval for the true spectral responsivity at wavelength *j*. Let

$$Q_{r}=\frac{1-u_{r}(y_{j})T_{\nu_{b}}^{\left(1\right)}}{1-u_{r}(c)T_{\nu_{c}}^{\left(2\right)}};$$ (6)

then

$$Y_{j}=Q_{r}{\tilde{y}}_{j}.$$

Since *u*_r_(*y_j_*) = *b*, *Q*_r_ is free of index *j*. Consequently, (*z*_1_, *z*_2_) = (*ỹ_j_ q*_1_, *ỹ_j_ q*_2_), where *q*_1_ and *q*_2_ are the 0.025 and 0.975 quantiles of the distribution of *Q*_r_. Note that *q*_1_ and *q*_2_ are the same for the entire wavelengths. To obtain the expanded uncertainty from the uncertainty interval (*ỹ_j_ q*_1_, *ỹ_j_ q*_2_), we note that in a 95 % *symmetric* interval of the form *x* ± *U*(*x*), the expanded uncertainty *U* (*x*) is half of the interval width. We write

$$({\tilde{y}}_{j}q_{1},{\tilde{y}}_{j}q_{2})=({\tilde{y}}_{j}-(1-q_{1}){\tilde{y}}_{j},{\tilde{y}}_{j}+(q_{2}-1){\tilde{y}}_{j}).$$ (7)

Since the interval (7) is not symmetric about *ỹ_j_* we use the maximum of (1 − *q*_1_) *ỹ_j_* and (*q*_2_ − 1) *ỹ_j_* as the expanded uncertainty of *ỹ_j_*. As a consequence, the relative expanded uncertainty of *ỹ_j_* based on the Supplement 1 method is given by

$$U_{r}^{*}=\max(1-q_{1},q_{2}-1).$$ (8)

The quantiles *q*_1_ and *q*_2_ are most conveniently estimated by use of a Monte Carlo approach. This involves generating a large number of realizations from the distribution of *Q*_r_ and determining *q*_1_ and *q*_2_ empirically. A single realization may be generated as follows:

1. Generate a realization of $T_{\nu_{b}}^{\left(1\right)}$ of a *t* random variable with *ν_b_* degrees of freedom.
2. Generate a realization of $T_{\nu_{c}}^{\left(2\right)}$ of a *t* random variable with degrees of freedom, independent of $T_{\nu_{b}}^{\left(1\right)}$.
3. Calculate *Q*_r_ as in (6).

We conduct a simulation study to compare the coverage probabilities of the uncertainty intervals constructed using *U*_r_ and $U_{r}^{*}$. The coverage probabilities depend on the values of *ν_b_* and *ν_c_*, as well as the true relative uncertainties of the monochromator-based spectralresponsivity measurements (denoted by σ_rm_) and of the adjustment factor (denoted by σ_rc_). Table 1 displays the coverage probabilities of the GUM and Supplement 1 intervals for various combinations of σ_rm_, σ_rc_, *ν_b_* and *ν_c_*. The simulation parameters used here are closely related to those we observed in our calibration experiments. The standard error in each entry of Table 1, based on the assumption of binomial distribution and 10000 simulation runs, is $\sqrt{0.95(1-0.95)/10000}=0.002$.

The above study indicates that the GUM intervals perform well even for the extreme cases where *ν_b_* = ∞ and *ν_c_* = 2. The Supplement 1 intervals are more conservative, which implies that $U_{r}^{*}$ is greater than *U*_r_ for the cases considered here. This is due to the symmetrization of the Supplement 1 intervals that expands the size of the intervals. As a consequence, we use *U*_r_ as the relative expanded uncertainty for the absolute responsivity in the final calibration report.

## 4. An Example

We use the data shown in Fig. 1 to illustrate the procedures described in Sec. 3. Measurements were made at 10 nm increments over wavelengths from 750 nm to 1800 nm by use of a monochromator-based system. A detailed uncertainty analysis for the system [1] yielded a relative standard uncertainty of 0.62 % with practically infinite degrees of freedom. Measurements were also made at wavelengths 850 nm, 1310 nm, and 1550 nm by use of a fiber-based system with a relative uncertainty of 0.25 %. The three pairs of spectral-responsivity measurements (*x_i_, y_i_*) (in A/W) at these wavelengths are (0.233, 0.2307), (0.7125, 0.7119), and (0.8073, 0.7986). The adjustment factor based on these three pairs of measurements is found to be *c* = 0.9934193, with the standard uncertainty *u* (*c*) = 0.00344334 or *u*_r_(*c*) = 0.346615 %. The line in Fig. 1 shows the normalized spectral responsivity.

To calculate the uncertainty of the normalized responsivity, we first calculate the effective degrees of freedom in (3) as

$$\nu ={\left(0.62^{2}+0.346615^{2}\right)}^{2}/(0.62^{4}/\infty +0.346615^{4}/2)\approx 35.$$

The relative expanded uncertainty is then given by

$$U_{r}=t_{0.975,35}\sqrt{0.0062^{2}+0.00346615^{2}}=1.44\%.$$

For comparison, the relative expanded uncertainty found by use of the Supplement 1 method is $U_{r}^{*}=1.90\%$ based on 500000 Monte Carlo samples.

## 5. Conclusion

In this paper we described procedures for calculating the normalized spectral responsivity and its relative expanded uncertainty. The normalization is based on the common spectral responsivity measurements made at some selected laser wavelengths. We compared two methods based on the GUM and Supplement 1 to the GUM for calculation of uncertainty. We used a simulation study to demonstrate that the uncertainty intervals constructed using the expanded uncertainty obtained from the GUM approach maintain the nominal level of 95 % for all the parameters we encountered in our experiments. Thus, the GUM-based expanded uncertainty is given in the calibration report.

## Figures and Tables

**Table 1:** Coverage probabilities of nominally 95 % GUM and Supplement 1 intervals

*σ*_rm_ (%)	*σ*_rc_ (%)	*ν_b_*	*ν_c_*	GUM	Sup 1
0.5	0.1	10	2	0.9515	0.9667
1	0.1	10	2	0.9471	0.9539
2	0.1	10	2	0.9500	0.9530
0.5	0.5	10	2	0.9509	0.9801
1	0.5	10	2	0.9567	0.9816
2	0.5	10	2	0.9527	0.9694
0.5	0.1	30	2	0.9497	0.9639
1	0.1	30	2	0.9501	0.9564
2	0.1	30	2	0.9489	0.9515
0.5	0.5	30	2	0.9447	0.9753
1	0.5	30	2	0.9493	0.9747
2	0.5	30	2	0.9510	0.9666
0.5	0.1	∞	2	0.9493	0.9628
1	0.1	∞	2	0.9522	0.9579
2	0.1	∞	2	0.9499	0.9529
0.5	0.5	∞	2	0.9451	0.9749
1	0.5	∞	2	0.9487	0.9751
2	0.5	∞	2	0.9504	0.9665
